# The biodiversity dose-response curve translates theory and practice from ecological restoration into research and clinical priorities for fecal microbiota transplantation

**DOI:** 10.3389/fmed.2022.1059148

**Published:** 2022-11-11

**Authors:** Matthew R. Orr

**Affiliations:** Department of Biology, Oregon State University-Cascades, Bend, OR, United States

**Keywords:** diet, biodiversity, interdisciplinary, dose-response, ecological restoration

## Abstract

Discoveries of the beneficial effects of gut microbiota have led to efforts to cultivate healthy gut flora to treat disease. The field of ecological restoration specializes on reestablishment of desired species in disturbed ecosystems, which suggests that it may be applicable to microbe restoration in the gut. Common language can lower barriers to interdisciplinary insights. Here I introduce the concept of a “biodiversity dose-response curve” to translate ideas from ecological restoration into research and clinical priorities for fecal microbiota transplantation (FMT). The curve is based on a relationship between ecosystem structure, measured as species diversity found in both nature and gut ecosystems, and ecosystem function, which are the measurable parameters that contribute to ecosystem and human health. I explain why the biodiversity dose-response curve may follow the ecological model of a “rivet-redundancy” relationship, in which the overlap of multiple organisms’ functional contributions to a system mask the impact of initial losses of diversity, but, at a certain level of loss, function declines sharply. (Imagine an airplane that flies with a few rivets missing, until it loses enough to fail.) The biodiversity dose-response curve indicates that seemingly healthy individuals may be suboptimal donors; it highlights the importance of recipient diet in FMT success; and it introduces the concept of “passive restoration” into the field of gut medicine. These insights, which may help to explain low success rates of FMT in the treatment of non-*Clostridium dificile* conditions, are less apparent in the absence of interdisciplinary integration.

## Introduction

The germ theory of disease established a foundation for a focus on pathogen inhibition. In recent decades, a more holistic paradigm of disease has emerged that, like germ theory, assigns a principal role to microbes in the etiology of illness. In contrast to germ theory, however, this new paradigm points to the cultivation of beneficial microbe species as a cure for disease. Fecal microbiota transplantation (FMT) is a promising approach for cultivating beneficial species in the gut microbiome. Until now, however, FMT has operated principally to eliminate *Clostridium dificile* infection [CDI (1)]. To date there exists no regulatory approval for non-CDI FMT (1, 2), and guidelines for FMT emphasize preventing side effects over promoting cures. A better understanding of the factors that promote establishment of beneficial biota in recipients is a priority because their scarcity associates with variety of non-CDI diseases including obesity, diabetes, cancer, and inflammatory bowel disease (IBD) (3).

The need to cultivate and support beneficial gut microbe communities for human health raises the question of whether preexisting approaches from other areas of science may be of assistance. For example, general ecological models of community assembly lead to predictions for the selection of effective fecal donors (4). More specifically, the field of ecological restoration seeks to assist the recovery of ecosystems that have been damaged, degraded, or destroyed (5). Because a human and its microbes can be considered close equivalents to an ecosystem (6), interventions that reconstitute healthy gut microbial communities for the improved health of their host could be viewed as an exercise in medical ecological restoration.

The field of ecological restoration is a few decades older than the field of microbiome medicine. Both rose quickly once the reliance of human wellbeing on intact ecosystems was recognized. In ecological restoration this is measured in the currency of “ecosystem services,” which are defined as benefits extracted by humans from nature (6). Rising recognition of ecosystem services, in concert with worsening degradation of the natural environment, led Wilson (7) to predict that the twenty first century would be “the era of restoration in ecology.” It is unlikely that Wilson made his prediction with gut medicine in mind, but he might as well have. To respond to the rise of worldwide conditions such as *C. dificile* infection, obesity, and inflammatory bowel disease (IBD), medical researchers, like their peers in ecology, have begun to manipulate the health-related ecosystem benefits provided by beneficial species (6). As in the case of natural ecosystems, such interventions raise questions about how to optimize their effectiveness.

The similarity of goals between ecological restoration and microbiome medicine present not only opportunities but also challenges common in interdisciplinary research, defined as “the synergistic combination of two or more disciplines to achieve one research objective” (8). Funding barriers, institutional organization, domain specificity, and conceptual and methodological divides commonly impede interdisciplinary efforts (9). Accordingly, this perspective piece seeks to highlight common conceptual ground between restoration ecology and gut medicine by translating a fundamental concept in ecology—structure-function curves—into a common medical concept—medicinal dose-response curves—*via* the idea of a “biodiversity dose-response curve.”

## The biodiversity dose-response curve

**FIGURE 1:**
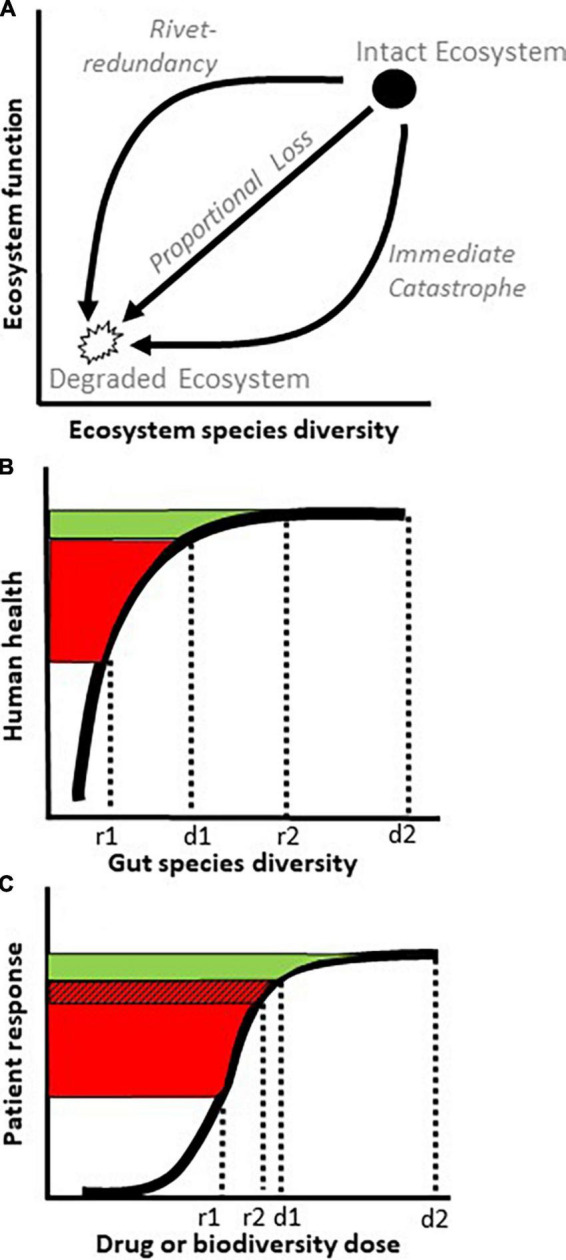
Curves relating species diversity with ecosystem function or host health. **(A)** Three ecological relationships between species diversity and ecosystem function. As an ecosystem transitions from an intact state with high diversity (filled circle) to a degraded state with low diversity (open polygon), its function (y axis) could decline *via* patterns of rivet-redundancy, proportional loss, or immediate catastrophe (arrows). **(B)** By virtue of its species redundancy, a rivet-redundancy relationship (thick curve), exhibits little increase in function (green) past a species diversity saturation point, d1. In terms of fecal microbiota transplantation (FMT), donors with species diversity d1 and d2 may exhibit roughly similar levels of health on the y axis (green), but incomplete engraftment of microbe diversity from donor d1 to recipient r1 risks a poor health outcome for the recipient (red). No similar risk exists from donor d2 to recipient r2 despite a similar or lower rate of engraftment. **(C)** In a medical dose-response relationship, drug efficacy flattens above dose d1 (green), similarly to rivet-redundancy in panel **(B)**. To increase the odds of FMT success, it may not only be beneficial to start with a high diversity donor (d2) but also to implement measures such as antibiotics, colon lavage, or an anti-inflammatory diet that reduce the risks of diversity loss during engraftment, shown here as a smaller gap between d1 and r2 than d1 and r1 and a smaller red hatched area than total red area [**(A,B)** adapted from (6)].

A principal goal in ecology is to understand how the species composition of an ecosystem influences its function. One approach is to quantify an ecosystem metric, like biological diversity, and see how it relates to an ecosystem property, like biomass production, nutrient uptake, or decomposition (10). This line of research has led to the conclusion that species diversity and ecosystem function most often follow a “rivet-redundancy” relationship [(10); Figure 1A], in which the system is robust to initial species losses, like an airplane losing a few rivets, but can collapse if too many species disappear, like an airplane losing enough rivets to fall apart midflight. The shape of this relationship is considered important for biological conservation because it mandates caution in assuming that a superficially healthy system can afford ongoing species losses (11).

Multiple lines of evidence support the hypotheses that, as in nature, gut ecosystems exhibit a rivet-redundancy relationship between microbe diversity and host health (Table 1). Although perhaps esoteric for scientists outside of ecology, the relationship in Figure 1B can be viewed analogously to a more familiar concept in medicine: a dose-response curve, leading to the concept of a “biodiversity dose-response curve” (Figure 1C). The biodiversity dose-response curve supports two insights for FMT. First, an apparently healthy donor is not necessarily an appropriate donor. Seemingly healthy donors who are close to a precipitous drop in function due to low microbiota diversity (d1 in Figures 1B,C) create a high likelihood of FMT failure if engraftment is incomplete (r1 in Figures 1B,C), and engraftment often is incomplete (12). Thus, to insure against FMT failure from partial engraftment, it may be important for potential donors to lie as far to the right on the biodiversity dose-response curve as possible, indicating a robust donor species diversity (d2 in Figures 1B,C).

**TABLE 1:** Lines of evidence supporting a rivet-redundancy relationship between species diversity (structure) and health (function) in the human gut.

Area	Evidence
General evidence for structure-function relationships in the gut	Functions of natural ecosystems including decomposition, nutrient flows, and biomass share analogs in the gut including metabolism, energy harvest, and body mass index.
	In both nature and the gut, ecosystem structure is commonly measured using metrics of species diversity.
	Gut species diversity correlates positively with health-related functions including obesity, IBD, diabetes, autism, allergies, asthma, cancer, and anorexia.
Specific evidence that gut structure-function relationships are rivet-redundant	Different humans harbor the same microbe-mediated metabolic pathways despite differences in the microbe species present, suggesting functional redundancy among species.
	Subsets of the complete microbiota perform the same functions as a complete microbiota in both humans and mice.
	Genes performing gut functions are commonly exchanged among gut microbes.
	Hosts would be unlikely to evolve an overreliance on a single microbe “keystone” species whose loss could jeopardize host fitness.

For details and citations see (6) pp. 80–81.

A second implication of the biodiversity dose-response curve is that high donor diversity should be complemented by treatments that optimize engraftment in recipients (shown as a smaller gap between d1 and r2 than d1 and r1 in Figure 1C). Engraftment success after FMT is comparable to the ecological priority of seedling establishment in natural ecosystems, which is often a limiting factor in restoration success (13). Seedlings may fail to establish due to poor site conditions such as degraded soil or undesired competitors (14). Accordingly, restoration ecologists tend to focus on “site preparation,” such as herbicides or watering, to remove unwanted competitors and improve seeding success (15). Analogous to the site prep of natural systems, site prep for FMT would include any gut intervention in a recipient that improves engraftment of donor biota, as discussed below.

## Discussion

As applications of FMT shift from pathogen removal for CDI to include the establishment of beneficial biota to treat non-CDI diseases, it is paramount to identify and prioritize the factors that best support shifts to a healthy gut biota among FMT recipients. What is the evidence that the features identified by the biodiversity dose-response curve—donor diversity and recipient site prep—merit priority in research and clinical practice of FMT? In terms of the importance of donor diversity, remarkably few studies have assessed its association with remission of symptoms after FMT, and studies that have are retrospective, lack replication, and/or are poorly controlled for confounding factors. Despite such shortcomings, evidence supporting donor diversity for FMT success is accumulating (16–18), but much more remains to be learned.

A meta-analysis consisting of 226 triads of donors, pre-FMT recipients, and post-FMT recipients across eight different disease types found that engraftment success associated with clinical success after FMT (12). In the same way that site preparation for establishment of beneficial species in natural systems often focuses on removing competing weeds, recipient site prep for successful engraftment in FMT includes measures such as antibiotics and bowel lavage that reduce dysbiotic taxa. In terms of experimental support for recipient site prep, engraftment success was found to associate more strongly with administration of pre-FMT antibiotics than it did with disease severity (19). In addition, patients with infectious conditions treated with antibiotics exhibited better engraftment than those with non-communicable conditions who did not receive antibiotics (12), although this finding was confounded by different disease conditions. Community ecology models together with suggestive but not significant clinical results also support the hypothesis that competition from a recipient’s resident microbes may reduce establishment of donor biota (4). More studies are needed to better understand the replicability of these findings and their relevance across different diseases.

Diet must also be considered for recipient site preparation. A gut disturbed by industrial, processed foods can be hostile to beneficial biota (6, 20). Viewed on the biodiversity dose-response curve, industrial diets may inhibit FMT by reducing the number and diversity, and therefore the “dose,” of donor biota that establish in the recipient (r1 vs. r2 in Figure 1C). In other words, poor quality or processed food may inhibit FMT success analogously to food-drug interactions that reduce drug activity or inhibit drug bioavailability (21). Effects of diet on FMT success may be more difficult to study than antibiotics and lavage due to the challenge of patient dietary compliance, which is analogous to the challenges of obtaining stakeholder compliance in ecological restoration (6, 20).

Perhaps because it is more difficult to control patient diets than it is to administer antibiotics, lavage, or even FMT, very few studies have examined dietary influences on FMT. In the only such study that I know of, subjects placed on an ulcerative colitis exclusion diet (UCED) plus FMT did not differ after 8 weeks from subjects on UCED alone or FMT alone (22). However, the UCED diet mandated yogurt, even though dairy is linked to UC (23). Moreover, UCED commenced at the same time as FMT, which may not be early enough to induce meaningful taxonomic shifts (24) or physiological responses, such as recovery of the intestinal mucus layer or intestinal epithelial cells (25), to prepare recipient guts for engraftment. Finally, the study’s low rate of patient responses is contradicted by a longer-term study in which FMT plus an anti-inflammatory diet that prohibited dairy was more effective than standard medical treatment in inducing both a clinical response and remission to UC (26). Much more needs to be done to better resolve effects of diet. A recent survey found that 71% of healthcare providers felt that diet was an important consideration for FMT, but they did not feel confident adding dietary protocols to FMT due to a lack of research to guide dietary advice (27).

Until proven otherwise, FMT without consideration of diet can be considered analogous to replanting sensitive species without removing the disturbances that facilitated noxious invaders in the first place (6). Viewed as such, ignoring diet in FMT violates a fundamental principle of ecological restoration: passive restoration, which removes disturbances such as livestock (analogous to removing fatty, sugary, and processed foods in the gut), must precede active restoration, which involves dynamic interventions such as weeding and herbicides (analogous to antibiotics and lavage) and species replantings (analogous to FMT). The principle of passive before active restoration is considered fundamental because active measures are less likely to succeed if the disturbances that caused degradation are permitted to persist.

A widespread failure to place passive restoration (i.e., diet) before active restoration (i.e., FMT) may help to explain a lack of evidence for long-term recipient microbiome changes after FMT in non-CDI diseases. A review of 24 non-CDI FMT research studies identified 19 studies that examined the duration of recipient microbiome changes. Of those, only three monitored recipients beyond 90 days post-treatment: one showed persistent changes for over a year and two reverted to no change after exhibiting an initial difference. Of the 16 studies that monitored for a shorter duration of 14–90 days, initial changes in recipient microbiomes either disappeared or became less significant over time in three studies (2). It is difficult to know how to interpret studies that do not demonstrate long term efficacy of FMT because failure to control for possible confounding effects of diet may increase the variability and reduce the magnitude of patient responses, leading to type II statistical errors. At least one study has attributed a failure to detect an FMT effect to low statistical power (4).

Restorationists tend to provide seedling support, such as by watering, for only a short duration of time due to practical considerations. If diet does influence engraftment of healthy microbiomes, research will be required to determine the degree to which short term dietary shifts are sufficient to support engraftment, or whether longer-term “lifestyle” changes before and/or after FMT are necessary. Such studies will require longer-term monitoring than most research on non-CDI diseases to date (2) as well as diet-without-FMT control groups, because changes in diet alone can be sufficient to alleviate IBD (20).

In summary, the biodiversity dose-response curve identifies factors likely to influence FMT success, beginning with diet as a form of passive restoration, followed by antibiotics or lavage for site prep, and finishing with high diversity donors to ensure sufficient engraftment above the threshold for system failure. These theoretical priorities are supported by early research into the beneficial effects of donor gut microbiome diversity (16) and recipient “site prep” [lavage, antibiotics (12, 19)], with little and contradictory evidence for diet (22, 26). Additional research is needed to better understand the extent to which these factors improve clinical success in FMT, which until now has been higher for CDI, in which the priority is pathogen removal, than it has for non-CDI diseases requiring the sustained establishment and restoration of beneficial biota (1).

## Data availability statement

The original contributions presented in this study are included in the article/supplementary material, further inquiries can be directed to the corresponding author.

## Author contributions

The author confirms being the sole contributor of this work and has approved it for publication.
